# *TCF7L2 *gene polymorphisms do not predict susceptibility to diabetes in tropical calcific pancreatitis but may interact with *SPINK1 *and *CTSB *mutations in predicting diabetes

**DOI:** 10.1186/1471-2350-9-80

**Published:** 2008-08-16

**Authors:** Swapna Mahurkar, Seema Bhaskar, D Nageshwar Reddy, Swami Prakash, G Venkat Rao, Shivaram Prasad Singh, Varghese Thomas, Giriraj Ratan Chandak

**Affiliations:** 1 Genome Research Group, Centre for Cellular and Molecular Biology, Uppal Road, Hyderabad, India; 2 Asian Institute of Gastroenterology, Punjagutta, Hyderabad, India; 3 Department of Gastroenterology, SCB Medical College, Cuttack, India; 4 Department of Gastroenterology, Calicut Medical College, Calicut, India

## Abstract

**Background:**

Tropical calcific pancreatitis (TCP) is a type of chronic pancreatitis unique to developing countries in tropical regions and one of its important features is invariable progression to diabetes, a condition called fibro-calculous pancreatic diabetes (FCPD), but the nature of diabetes in TCP is controversial. We analysed the recently reported type 2 diabetes (T2D) associated polymorphisms in the *TCF7L2 *gene using a case-control approach, under the hypothesis that *TCF7L2 *variants should show similar association if diabetes in FCPD is similar to T2D. We also investigated the interaction between the *TCF7L2 *variants and N34S *SPINK1 *and L26V *CTSB *mutations, since they are strong predictors of risk for TCP.

**Methods:**

Two polymorphisms rs7903146 and rs12255372 in the *TCF7L2 *gene were analyzed by direct sequencing in 478 well-characterized TCP patients and 661 healthy controls of Dravidian and Indo-European ethnicities. Their association with TCP with diabetes (FCPD) and without diabetes was tested in both populations independently using chi-square test. Finally, a meta analysis was performed on all the cases and controls for assessing the overall significance irrespective of ethnicity. We dichotomized the whole cohort based on the presence or absence of N34S *SPINK1 *and L26V *CTSB *mutations and further subdivided them into TCP and FCPD patients and compared the distribution of *TCF7L2 *variants between them.

**Results:**

The allelic and genotypic frequencies for both *TCF7L2 *polymorphisms, did not differ significantly between TCP patients and controls belonging to either of the ethnic groups or taken together. No statistically significant association of the SNPs was observed with TCP or FCPD or between carriers and non-carriers of N34S *SPINK1 *and L26V *CTSB *mutations. The minor allele frequency for rs7903146 was different between TCP and FCPD patients carrying the N34S *SPINK1 *variant but did not reach statistical significance (OR = 1.59, 95% CI = 0.93–2.70, P = 0.09), while, *TCF7L2*variant showed a statistically significant association between TCP and FCPD patients carrying the 26V allele (OR = 1.69, 95% CI = 1.11–2.56, P = 0.013).

**Conclusion:**

Type 2 diabetes associated *TCF7L2 *variants are not associated with diabetes in TCP. Since, *TCF7L2 *is a major susceptibility gene for T2D, it may be hypothesized that the diabetes in TCP patients may not be similar to T2D. Our data also suggests that co-existence of *TCF7L2 *variants and the *SPINK1 *and *CTSB *mutations, that predict susceptibility to exocrine damage, may interact to determine the onset of diabetes in TCP patients.

## Background

Pancreatitis is generally believed to be a disease where pancreas is injured by enzymes that are normally secreted by the acinar cells. Chronic Pancreatitis (CP) is a continuing or relapsing inflammatory process of pancreas leading to exocrine and/or endocrine insufficiency. Tropical calcific pancreatitis (TCP) is a type of CP unique to developing countries in tropical region [1]. An important feature of TCP is its consistent progression to diabetes, commonly known as fibro-calculous pancreatic diabetes (FCPD) [1,2]. FCPD is thought to be a type of diabetes secondary to TCP, resulting from destruction of beta-cell mass in the pancreas [3]. However, several studies have shown partial preservation of beta-cell mass [3] and evidence of insulin resistance to a similar degree as seen in type 2 diabetes (T2D) patients [4], suggesting that the diabetes in FCPD could be type T2D, while others have not found insulin resistance to be a major factor in FCPD [5]. It was believed earlier that diabetes specific complications do not occur in FCPD [6], but prevalence of retinopathy, [7] nephropathy and neuropathy [8] in FCPD patients has been reported to be no different from matched group of patients with T2D. Similarly, although the diabetes is severe and insulin requiring in both FCPD and type 1 diabetes (T1D), FCPD patients rarely develop ketoacidosis, in contrast to the T1D patients, who are ketosis prone [9,10].

Mutations in the serine protease inhibitor, Kazal type 1 (*SPINK1*) [11-15], cystic fibrosis transmembrane regulator (*CFTR*) [16], cathepsin B (*CTSB*) [17] and recently, chymotrypsin C (*CTRC*) [18] genes have been identified to be associated with TCP but they mostly associate with pancreatic exocrine dysfunction. No study has yet investigated the genetic basis of diabetes in TCP and FCPD. Based on the suggestive linkage of T2D to chromosome 10q, a microsatellite, DG10S478, within intron 3 of transcription factor 7-like 2 (*TCF7L2*) gene was found to be strongly associated with T2D [19]. An association of a variant of the gene, rs7903146 along with other SNPs in linkage disequilibrium with this polymorphism was first reported in Islandic, Danish and in the US cohort [19]. Subsequently this association was replicated in other populations like Indian [20,21], French [22], U.K [23] and Finnish populations [24], and these variants account for the highest T2D risk confirmed to date [25]. *TCF7L2 *gene variants have also been proposed to play important role in T1D because of its effects on blood glucose homeostasis [26]; however a recent study failed to find any association and age-of-onset effect of T1D with rs7903146 SNP in *TCF7L2 *gene [27]. This suggested that a T2D mechanism mediated by polymorphisms in *TCF7L2 *does not participate in the etiology of T1D, thus susceptibility factors for T2D could be different from those involved in T1D. Hence, investigating a known susceptibility factor for T1D or T2D can help in understanding the type and mechanism of diabetes in FCPD patients.

As the type of diabetes in FCPD is not clearly understood, we used association of *TCF7L2 *variants with T2D as a marker to decipher the type of diabetes in FCPD. Since there are suggestions that TCP is the pre-diabetic stage of FCPD, we also analyzed the association of *TCF7L2 *polymorphisms in TCP patients. We hypothesized that we would observe an association of variants in the *TCF7L2 *gene with FCPD, if diabetes in these patients is T2D. Since diabetes in TCP is also thought to be due to destruction of endocrine pancreatic cells secondary to destruction of exocrine pancreas, we investigated the interaction between the *TCF7L2 *variants and N34S *SPINK1 *and L26V *CTSB *mutations and explored whether presence of *TCF7L2 *variants in patients with these mutations predisposes them to FCPD.

## Methods

### Patients and controls

478 unrelated individuals (320 males and 158 females) were diagnosed as TCP (n = 286) or FCPD (n = 192) patients based on the established WHO criteria [28]. Of these, 333 patients were of Dravidian ethnicity and 145 belonged to Indo-European ethnicity. Six hundred and sixty one age matched individuals (332 males and 329 females) comprising of 259 Dravidians and 402 Indo-Europeans without any complaints and evidence of pancreatitis were included as controls [17,20]. Both patients and the controls filled a detailed questionnaire and signed a written informed consent for genetic analysis. The Institutional Ethics Committee of all the institutes approved the study following the Indian Council of Medical Research guidelines for research on human subjects.

### Genetic analysis

Genomic DNA from all the patients and healthy volunteers were utilized for this study. Primers, amplifying segments of *TCF7L2 *gene harboring SNPs rs7903146 and rs12255372 were adopted from our earlier study [20]. PCR products were purified and sequenced individually on both the strands using Big-dye terminator cycle sequencing ready kit (Applied Biosystems, Foster City, CA) on an ABI3730 Genetic Analyzer (Applied Biosystems Foster City, CA). In case of unclear sequence data, we repeated direct sequencing under various conditions until the genotype was determined correctly. Ten percent of the genotyping results were validated on tetra primer based analysis for the 2 SNPs [20] and no discrepancy was observed.

### Statistical analysis

The allele and genotype frequencies were calculated for the SNPs (table 1) in cohorts of both ethnicities separately as well as together and to analyze deviation from the Hardy-Weinberg equilibrium, observed and expected genotype frequencies were compared by Markov simulation based goodness of fit test [29]. Chi-square test was used to analyze the statistical significance of the difference in allelic distribution of various polymorphisms in patients and controls (DeFinitte; ). For assessing the overall significance irrespective of ethnicities, the meta-analysis statistic was used and the forest plots were generated under the fixed effect model using Comprehensive Meta Analysis  software version 2.2.046 and the Q test was used to test for homogeneity of groupings [30]. The whole cohort was dichotomized initially based on the presence or absence of N34S *SPINK1 *and L26V *CTSB *mutations and then the two groups were subdivided into TCP and FCPD patients and distribution of *TCF7L2 *variants was compared between them. Unless indicated specifically, a p-value of 0.05 was considered significant in all the analyses. This study with random selection of patients and controls has 80% power to detect an effect with an OR as low as 1.3 at α = 0.05 and 95% power at the OR of 1.46, which was identified in our earlier study on T2D subjects [20].

**Table 1:** Allelic and genotypic frequencies for the *TCF7L2 *variants in TCP patients and controls of different ethnic groups

SNP (NCBI 36.2^&^)	Allele	Dravidian		Indo-Europeans		Total		Genotype	Dravidian^@^		Indo-Europeans^@^		Total^@^	
		Patients	Controls	Patients	Controls	Patients	Controls		Patients	Controls	Patients	Controls	Patients	Controls
		n = 333	n = 259	n = 145	n = 402	n = 478	n = 661		n = 333	n = 259	n = 145	n = 402	n = 478	n = 661
rs7903146 (114748339)	C	0.71	0.70	0.72	0.71	0.73	0.71	CC	175 (52.6)	130 (50.2)	78 (53.8)	207 (51.5)	253 (53.0)	337 (51.0)
								CT	126 (37.8)	104 (40.2)	53 (36.6)	160 (39.8)	179 (37.3)	264 (39.9)
	T	0.29	0.30	0.28	0.29	0.27	0.29	TT	32 (9.6)	25 (9.7)	14 (9.7)	35 (8.7)	46 (9.6)	60 (9.1)
														
		n = 332	n = 180	n = 144	n = 402	n = 476	n = 582		n = 332	n = 180	n = 144	n = 402	n = 476	n = 582
														
rs12255372 (114798892)	G	0.77	0.78	0.78	0.78	0.77	0.78	GG	201 (60.5)	108 (60.0)	88 (61.1)	244 (60.7)	289 (60.7)	352 (60.5)
								GT	110 (33.1)	64 (35.6)	48 (33.3)	135 (33.6)	158 (33.2)	199 (34.2)
	T	0.23	0.22	0.22	0.22	0.23	0.22	TT	21 (6.3)	8 (4.4)	8 (5.6)	23 (5.7)	29 (6.1)	31 (5.3)

SNP, single nucleotide polymorphism; n, number of individuals; ^&^, Chromosome position according to National Centre for Biotechnology Information (NCBI), Build 36.2, contig accession number NT 030059.12; ^@^, values in the parentheses indicate percentage genotype frequency

## Results and discussion

The two polymorphisms rs7903146 and rs12255372 in the *TCF7L2 *gene, reported to be most strongly associated with T2D, were analyzed in a cohort of TCP and FCPD patients and controls belonging to Dravidian and Indo-European ethnicities. It is believed by most workers in the field that FCPD is the logical end point of TCP and enough evidence exists to suggest that TCP is the pre-diabetic stage of FCPD [3]. Thus, we also analyzed the association of *TCF7L2 *variants in the entire cohort irrespective of their diabetic status. In addition, clinical presentation is known to be variable for FCPD [1,2]. Most of the patients present with pain abdomen and evidence of pancreatitis and subsequently develop diabetes at a later stage; a small proportion present with diabetes and are detected to have pancreatic stones and calcification on subsequent investigations [1,2]. It may be surmised that additional diabetes susceptibility gene may account for the earlier phenotype of diabetes. Hence, an attempt was also made to dichotomize the cohort into TCP and FCPD to investigate whether FCPD patients have an additional risk due to *TCF7L2 *polymorphisms. We also investigated whether co-inheritance of *TCF7L2 *variants with the *SPINK1 *and *CTSB *mutations predisposes these patients to develop diabetes.

**Figure 1:**
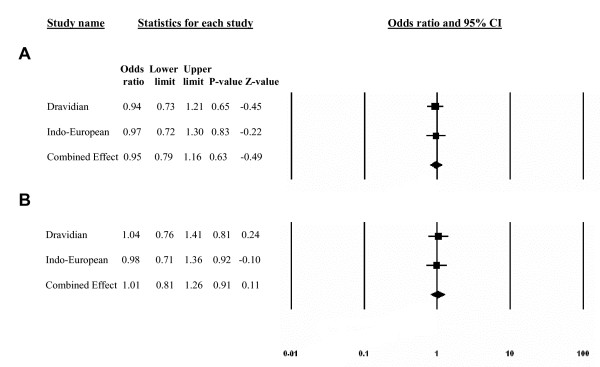
**Meta-analysis for association of TCF7L2 variants with TCP**. Forest plot showing results of meta analysis. Odds ratio for each study is represented by a block bounded by its confidence interval; Combined effect for the two studies has been calculated using fixed effect model; A, rs7903146; B, rs12255372.

Both the polymorphisms followed Hardy-Weinberg equilibrium (p > 0.05) and on comparing the allele frequencies within the ethnic groups, Dravidian patients vs Dravidian controls and Indo-European patients vs Indo-European controls, no significant differences were seen (table 1), neither did the genotype relative risk differ significantly between patients and controls (table 2). A meta-analysis of all cases and control subjects from both ethnicities, showed similar results for both SNPs in *TCF7L2 *gene [(95%CI, 0.95–1.16; P = 0.63, Cochran's Q = 0.0092, P = 0.92 for rs7903146) and (95%CI, 1.01–1.29; P = 0.92, Cochran's Q = 0.0482, P = 0.83 for rs12255372)] (fig 1). In order to explore the possibility of association of *TCF7L2 *variants with FCPD, the analysis was carried out in TCP patients with diabetes (FCPD) and those without diabetes separately. Allele and genotypic frequencies did not differ significantly, between TCP patients and controls, FCPD patients and controls and between TCP and FCPD patients, suggesting lack of statistically significant association of *TCF7L2 *polymorphisms with FCPD (table 3).

**Table 2:** Estimates of the genotype and allele relative risks for the *TCF7L2 *variants in the cases and controls based on ethnicity

	SNP*	Het OR (95% CI)	P	Hom OR (95% CI)	P	^&^OR (95% CI)	P
All cases vs all controls	rs7903146	0.90 (0.70–1.15)	0.40	1.02 (0.67–1.55)	0.92	0.96 (0.80–1.16)	0.70
	rs12255372	1.03 (0.80–1.34)	0.80	0.88 (0.52–1.49)	0.62	0.98 (0.80–1.21)	0.88
DR cases vs DR controls	rs7903146	0.90 (0.64–1.27)	0.55	0.95 (0.54–1.68)	0.86	0.94 (0.73–1.21)	0.65
	rs12255372	0.92 (0.63–1.36)	0.69	1.41 (0.60–3.30)	0.42	1.04 (0.76–1.41)	0.81
IE cases vs IE controls	rs7903146	0.86 (0.57–1.30)	0.48	1.06 (0.54–2.08)	0.86	0.96 (0.71–1.29)	0.79
	rs12255372	0.99 (0.65–1.48)	0.95	0.96 (0.42–2.23)	0.93	0.98 (0.71–1.36)	0.92

SNP, single nucleotide polymorphism; *baseline genotype at rs7903146-CC, rs12255372-GG; Het OR & Hom OR, genotype relative risk (GRR) for heterozygotes and homozygotes respectively (GRR was calculated by comparing with the baseline genotype); ^&^, allelic OR; P, P value; DR, Dravidians; IE, Indo-European

**Table 3:** Estimates of the genotype and allele relative risks for the *TCF7L2 *variants in the cases and controls based on clinical diagnosis

	SNP*	Het OR (95% CI)	P	Hom OR (95% CI)	P	^&^OR (95% CI)	P
TCP (n = 286) vs Controls	rs7903146	0.95 (0.71–1.28)	0.74	0.98 (0.59–1.61)	0.94	0.97 (0.78–1.21)	0.81
	rs12255372	0.92 (0.68–1.26)	0.62	1.31 (0.72–2.36)	0.38	1.03 (0.81–1.31)	0.79
FCPD (n = 192) vs Controls	rs7903146	0.82 (0.58–1.16)	0.27	1.08 (0.62–1.87)	0.78	0.95 (0.74–1.22)	0.69
	rs12255372	1.03 (0.73–1.46)	0.86	0.89 (0.41–1.92)	0.76	0.99 (0.75–1.31)	0.94
TCP vs FCPD	rs7903146	0.86 (0.58–1.28)	0.47	1.10 (0.58–2.08)	0.76	0.98 (0.73–1.30)	0.87
	rs12255372	1.11 (0.75–1.65)	0.59	0.68 (0.30–1.55)	0.36	0.96 (0.70–1.31)	0.79

SNP, single nucleotide polymorphism; TCP, tropical calcific pancreatitis; FCPD, fibro-calculous pancreatic diabetes; *baseline genotype at rs7903146-CC, rs12255372-GG; Het OR & Hom OR, genotype relative risk (GRR) for heterozygotes and homozygotes respectively (GRR was calculated by comparing with the baseline genotype); ^&^, allelic OR; P, P value

Association analysis of the total cohort after dichotomization based on N34S *SPINK1 *and L26V *CTSB *mutation status showed comparable allele and genotype frequencies for rs7903146 in both groups, indicating that co-existence of these variants does not increase the risk of developing diabetes in these patients (table 4). However, the minor allele frequency for rs7903146 was different between TCP and FCPD patients carrying the N34S *SPINK1 *variant but did not reach statistical significance (OR = 1.59, 95% CI = 0.93–2.70, P = 0.09). Interestingly, similar analysis using L26V *CTSB *variant showed a statistically significant association between TCP and FCPD patients carrying the mutant allele compared to those without the variant (OR = 1.69, 95% CI = 1.11–2.56, P = 0.013) (table 4). Similar results were obtained on analysis of the rs12255372 variant in *TCF7L2 *gene (data not presented). This suggests that co-existence of *TCF7L2 *variants and the variants predicting susceptibility to exocrine damage may interact to determine the onset of diabetes in TCP patients. However, this may need to be replicated in larger sample size since there is a possibility of a chance association due to small sample size.

**Table 4:** Association of *TCF7L2 *variant rs7903146 on dichotomization of the patient cohort based on N34S *SPINK1 *and L26V *CTSB *mutation status

	**N34S *SPINK1***				**L26V *CTSB***			
						
	**Wild**	**Mutant**			**Wild**	**Mutant**		
**rs7903146**	**MAF**	**MAF**	**OR (95% CI)**	**P**	**MAF**	**MAF**	**OR (95% CI)**	**P**
**Total Cohort**	(n = 307)	(n = 148)			(n = 165)	(n = 236)		
	0.29	0.28	0.95 (0.70–1.30)	0.75	0.28	0.28	1.01 (0.74–1.38)	0.95
**FCPD**	(n = 129)	(n = 49)			(n = 65)	(n = 78)		
	0.31	0.35	1.13 (0.69–1.85)	0.63	0.28	0.36	1.42 (0.86–2.36)	0.17
**TCP**	(n = 178)	(n = 99)			(n = 100)	(n = 158)		
	0.27	0.24	0.87 (0.58–1.29)	0.48	0.28	0.24	0.83 (0.55–1.24)	0.36
**FCPD vs TCP**								
OR	1.22 (0.85–1.73)	1.59 (0.93–2.70)			0.98 (0.60–1.61)	1.69 (1.11–2.56)		
P	0.27	0.09			0.95	0.013*		

TCP, tropical calcific pancreatitis; FCPD, fibro-calculous pancreatic diabetes; MAF, Minor allele frequency; OR, odds ratio; CI, confidence interval; P, p value;

We and others have earlier replicated the strong association of *TCF7L2 *variants with T2D in Indian population and provided evidence of its likely role in the pathogenesis of T2D by influencing both insulin secretion and insulin resistance [20]. According to accelerator hypothesis, T1D and T2D may share a common etiology of hyperglycemia-induced beta cell damage but T1D may have the added effects of autoimmunity [31]. However, the lack of association of *TCF7L2 *with T1D, as shown by Field et al., does not support the model of a shared major causal pathway in T2D and T1D [32]. Hence the genes that determine susceptibility to T1D must be different from the susceptibility genes of T2D. This has important implications for diabetes in TCP since overlapping features of T1D and T2D are observed in FCPD. As the overall evidence for association of *TCF7L2 *gene variants exceeds genome-wide significance criteria (10^-5^) and clearly establishes *TCF7L2 *as a T2D susceptibility gene of substantial importance in majority of populations world-wide [33] including Indian population [20], it is less likely that T2D may have a susceptibility factor stronger than *TCF7L2*. A lack of association of *TCF7L2 *with TCP or FCPD observed in our study, may suggest a role for genes other than *TCF7L2 *to be predictive of susceptibility to T2D. Since there is debate about the type of diabetes in TCP and FCPD, the lack of association with *TCF7L2*, the gene most strongly associated with T2D may suggest that the diabetes in TCP patients does not have similar features as T2D.

## Conclusion

As *TCF7L2 *is a major susceptibility gene for T2D, a lack of association of *TCF7L2 *variants with TCP or FCPD observed in our study suggests that T2D associated *TCF7L2 *variants are not associated with diabetes in TCP or the diabetes in TCP patients may not be similar to T2D. Thus, although the variations in *TCF7L2 *increase the risk for T2D and may affect insulin secretion, they do not alter susceptibility to FCPD, the diabetes in TCP patients. However, co-inheritance of the *TCF7L2 *variants with the pancreatitis associated susceptibility variants in *SPINK1 *and *CTSB *genes may predict the development of diabetes in these patients, but these observations need to be confirmed independently.

## Competing interests

The authors declare that they have no competing interests.

## Authors' contributions

SM did all the genotyping, statistical analysis and wrote the first draft of the manuscript. SB and SP assisted in the genotyping and statistical analysis whereas DNR, GVR, SPS and VT were involved in the recruitment of the patients and controls. GRC conceptualized the study, supervised the results and finalized the manuscript. All the authors have gone through the manuscript and have consented to the final manuscript.

## Pre-publication history

The pre-publication history for this paper can be accessed here:


